# MicroRNA-8 promotes robust motor axon targeting by coordinate regulation of cell adhesion molecules during synapse development

**DOI:** 10.1098/rstb.2013.0517

**Published:** 2014-09-26

**Authors:** Cecilia S. Lu, Bo Zhai, Alex Mauss, Matthias Landgraf, Stephen Gygi, David Van Vactor

**Affiliations:** 1 Department of Cell Biology, Harvard Medical School, Boston, MA 02115, USA; 2 Program in Neuroscience, Harvard Medical School, Boston, MA 02115, USA; 3 Okinawa Institute of Science and Technology Graduate University, Okinawa, Japan; 4 Department of Zoology, University of Cambridge, Cambridge, UK; 5 Max Planck Institute of Neurobiology, Martinsried, Germany

**Keywords:** synapse development, *Drosophila* neuromuscular junction, microRNA, miR-8, Fasciclin III, Neuroglian

## Abstract

Neuronal connectivity and specificity rely upon precise coordinated deployment of multiple cell-surface and secreted molecules. MicroRNAs have tremendous potential for shaping neural circuitry by fine-tuning the spatio-temporal expression of key synaptic effector molecules. The highly conserved microRNA miR-8 is required during late stages of neuromuscular synapse development in *Drosophila*. However, its role in initial synapse formation was previously unknown. Detailed analysis of synaptogenesis in this system now reveals that miR-8 is required at the earliest stages of muscle target contact by RP3 motor axons. We find that the localization of multiple synaptic cell adhesion molecules (CAMs) is dependent on the expression of miR-8, suggesting that miR-8 regulates the initial assembly of synaptic sites. Using stable isotope labelling *in vivo* and comparative mass spectrometry, we find that miR-8 is required for normal expression of multiple proteins, including the CAMs Fasciclin III (FasIII) and Neuroglian (Nrg). Genetic analysis suggests that Nrg and FasIII collaborate downstream of miR-8 to promote accurate target recognition. Unlike the function of miR-8 at mature larval neuromuscular junctions, at the embryonic stage we find that miR-8 controls key effectors on both sides of the synapse. MiR-8 controls multiple stages of synapse formation through the coordinate regulation of both pre- and postsynaptic cell adhesion proteins.

## 1. Introduction

Micro(mi)RNAs have emerged as versatile regulators of gene expression capable of fine-tuning the expression patterns and levels of many proteins through multiple post-transcriptional mechanisms [1]. Sequence analysis of the expressed genome in many metazoan species reveals hundreds of predicted mRNA targets for miRNA regulation [2–6]. Although bioinformatics alone cannot identify functionally relevant miRNA targets, sequence analysis suggests that over 60% of human protein-coding genes are under some degree of selective pressure to maintain pairing with miRNAs [7]. In addition to direct targeting of downstream mRNAs, miRNA can control gene expression of secondary targets through multiple classes of intermediary regulators (i.e. transcription factors, RNA-binding proteins, etc.). This suggests that a complex and potentially dynamic gene network underlies the functions of many miRNAs. However, the identification and *in vivo* analysis of the functionally relevant target gene networks orchestrated and controlled by miRNAs remains a significant challenge in the field.

The striking expression of many miRNA in the nervous system [8–11] and an early wave of functional studies for a handful of brain-enriched candidates [12,13] reveal that miRNA genes participate in the formation, maintenance and activity-dependent remodelling of synapses [14]. Prior to the onset of neural activity, axon guidance and synaptogenesis follow stereotyped developmental programmes that specify neuronal identity and establish chemical affinity between synaptic partners through the control of gene expression [15]. One excellent system with which to study the relationship between genetic regulatory networks and synaptogenesis is the developing neuromuscular junction (NMJ) of *Drosophila melanogaster* (*Drosophila*). In *Drosophila* embryos and larvae, each abdominal hemisegment of the animal contains a stereotyped pattern of 30 muscles innervated by approximately 34 motoneurons, each individually identifiable by its size, shape and expression of molecular markers [16–19]. A rich network of molecular pathways and cell-surface receptors required for *Drosophila* NMJ formation, maintenance and homeostasis has been defined by a community of investigators [20–22], setting the stage for studying the layers of regulatory mechanism that are required to achieve normal synapse development in this system.

We recently identified the conserved *Drosophila* miRNA miR-8 in a screen for modulators of a signalling pathway that controls multiple phases of axon guidance and synaptogenesis. At the mature larval NMJ, *Drosophila* miR-8 is required for the morphological expansion of the synapse required to match the substantial growth of target muscles during larval development but is downregulated by synaptic stimulation to allow activity-dependent synaptogenesis [23–25]. Other studies of miR-8 and its vertebrate homologues (miR-141/200) showed that this conserved miRNA family contributes to the regulation of diverse biological processes from neurodegeneration, limb/wing patterning and osmotic stress response to fat metabolism in the control of body size [26–29]. In each of these contexts, one key target gene was identified that could account for the majority of miR-8 loss- or gain-of-function mutant defects. Here, we examined the role of miR-8 at early stages of NMJ development. Combined with analysis of new downstream genes identified through differential proteomic profiling of wild-type and mutant tissue (see §3*c*), we find that miR-8 regulates an early stage of synapse development via multiple downstream effector genes. Using stable isotope labelling and comparative mass spectrometry, we found that miR-8 is required for embryonic expression of the synaptic immunoglobulin superfamily cell adhesion molecules (IgCAMs) Fasciclin III (FasIII) and Neuroglian (Nrg). We show that the deployment of FasIII and Nrg in a subset of motor axons and their target muscles is dependent on miR-8, suggesting that miR-8 regulates the initial assembly of synaptic sites at the time of initial neuron-target muscle contact. Finally, genetic analysis in the *Drosophila* embryo supports a model where Nrg and FasIII cooperate to promote synapse formation downstream of miR-8.

## 2. Material and methods

### (a) Fly strains

We generated *miR-8^Δ/Δ^* using FRT/FLP targeted deletion of *miR-8* flanked by P{XP}d01682 and PBac{WH}f05125 [30,31]. Two-sided PCR was used to isolate the recombinant, and genomic PCR spanning the breakpoints confirmed the deletion. The gross phenotypes of *miR-8^Δ/Δ^* including leg and wing deformation as well as NMJ defects are comparable to another null allele *miR-8^Δ^*^2^ (a gift from S. Cohen, [23,26]). The following *nrg* and *fasIII* alleles were used: *nrg^14^/FM7c* (also known as *nrg^1^*) is a null, *nrg^17^/FM7c* (also known as *nrg^2^*) is a strong hypomorph [32], and the amorphic *fasIII^A142^*/*CyO* from the Berkeley Drosophila Genome Project collection was inserted by PBac{5HPw+} [33]. Lethal mutations/insertions were kept over *FM7c*, *CyO* and *TM6B* or *TM3* balancer chromosomes that are additionally marked with *twi-GAL4::UAS-EGFP*, *Dfd-EYFP* or *wg-lac*Z which express GFP, YFP or β-galactosidase during embryogenesis to facilitate identification of embryos harbouring homozygous mutant alleles. As wild-type controls, strains isogenic *w^1118^*and *islet-*τ*-mycGFP* [34] were used. Fly stocks *Elav-GAL4, how^24B^-GAL4*, *UAS-nrg^180^*, and all of those mentioned above were obtained from Bloomington Drosophila Stock Center and the Exelixis Collection at Harvard Medical School. All strains were maintained and crossed at 25°C.

### (b) Immunohistochemistry and image analysis

Embryos were immunostained according to standard procedures [35], dissected and mounted in 70% glycerol (DIC) or SlowFade Gold anti-fade reagent (Invitrogen). Primary antibodies against the following molecules were used: monoclonal mouse anti-FasII (1D4, 1: 4), anti-FasIII (7G10, 1 : 5), anti-Nrg (BP104, 1 : 10) from Developmental Studies Hybridoma Bank, Iowa City, USA (DSHB); rabbit anti-HRP 1 : 1500 (Jackson ImmunoResearch), rabbit anti-GFP 1 : 500 (Abcam), rabbit β-galactosidase 1 : 5000 (Cappel). HRP-conjugated secondary antibodies were purchased from Jackson ImmunoResearch; DAB Peroxidase Substrate Kit was from Vector Labs; Alexa Fluor 488-, 546-conjugated secondary antibodies and Alexa Fluor 633 phalloidin for muscle F-actin staining were from Invitrogen.

DIC images were taken with 63× (1.4 N.A.) Plan Apochromat objective (Nikon) in oil and a Spot camera mounted on a Zeiss Axio Plan II microscope operated by Spot Imaging Solution software. Laser confocal images were acquired using Nikon TE2000 with C1 point scanning and Zeiss LSM510 META confocal microscopes with 40× (0.95 N.A.) objective in oil, 1.5× digital zoom, and shown as maximal projections of confocal image stacks. We used the NIH ImageJ program to measure axon length and compute synaptic coverage area from confocal image stacks. For quantitative imaging analysis, we used Alexa Fluorophores with excitation/emission characteristics compatible with the wavelengths of lasers and META spectral emission detectors installed in the Zeiss LSM510 system to minimize signal bleed-through between any two channels from overlapping fluorescence emission spectra. During image acquisition, we calibrated the settings to image below the saturating level of fluorescence intensity across different specimens and applied the same settings to pairs of experiment and control genotypes. Quantification of immunofluorescence was performed by integrating the mean signal intensity within regions of interest that was identical in each optical slice over the thickness of confocal image stacks. We computed average values obtained from all embryos of the same genotype on the same slide and compared those values between different genotypes prepared in parallel on the same day.

### (c) DiI fill of RP3 motor neurons

All embryos were raised at 25°C, dissected on poly-lysine-coated coverslips at 15 h after egg laying (AEL) and fixed in 3.7% formaldehyde (less than 10 min). A total of 2 mg ml^−1^ DiI was backfilled into sharp electrodes and electrode shafts were further backfilled with 0.2 M LiCl. DiI was injected into RP3 motor neurons in abdominal segments A2–A6 by application of a depolarizing current of 0.4–1 nA using an Iontophoretic Dye Marker amplifier, D380 (Digitimer, UK). Muscles were perforated and counterstained with Alexa Fluor 647 phalloidin overnight at 8°C. Specimens were imaged with 63× (1.2 N.A.) water immersion objective (Olympus) and a spinning disc (CSU-22; Yokagawa) confocal field scanner mounted on an Olympus BX51WI microscope, operated by Metamorph (7.1) software (Molecular Devices). Optical slices were acquired at 300 nm intervals with effective pixel dimensions 210 nm × 210 nm controlled by a single objective Piezo drive (Physik Instruments).

### (d) *In vivo* SILAC

Five milligrammes of 0–10 h-old *Drosophila* embryos was collected and transferred onto Whatman filter paper over a layer of cotton to hatch in a humidity-saturated chamber at 25°C. The F_1_ larvae were fed with liquid fly food and fresh yeast paste made from Lys^−^/Arg^−^ double auxotroph *Saccharomyces cerevisiae* strain 3681 (gift from A. Rudner, University of Ottawa) grown to saturation in lysine and arginine drop-out YNB media (Difco) supplemented with either light isotope l-lysine and l-arginine (Sigma) or heavy l-[^13^C_6_]-lysine and l-[^13^C_6_]-arginine (Cambridge Isotope Laboratories). The culture and labelling of yeast was carried out using the following procedures as described in [36]. Upon hatching, the F_1_ adult flies were transferred to new egg-laying cages supplied with light or heavy isotope labelled fresh yeast paste. Twenty milligrammes of F_2_ embryos was collected and homogenized in RIPA buffer. Control and *miR-8* mutant embryo lysates were standardized using the Bradford assay (Pierce Biotechnology) and combined at a 1 : 1 ratio using 150 μg protein from each sample (300 μg total), boiled in SDS-PAGE sample buffer, resolved on a 4–15% Tris-glycine gel and stained with Coomassie Blue (BioRad Laboratories). A single gel lane was excised, divided vertically into 12 sections and each section excised and subjected to in-gel trypsin digestion. The tryptic peptides were extracted from the gel and analysed by liquid chromatography tandem mass spectrometry followed by the identification and quantification of peptides (see the electronic supplementary material).

## 3. Results

### (a) Genetic deletion of miR-8 causes subtle innervation defects at the embryonic neuromuscular junction

**Figure 1.**
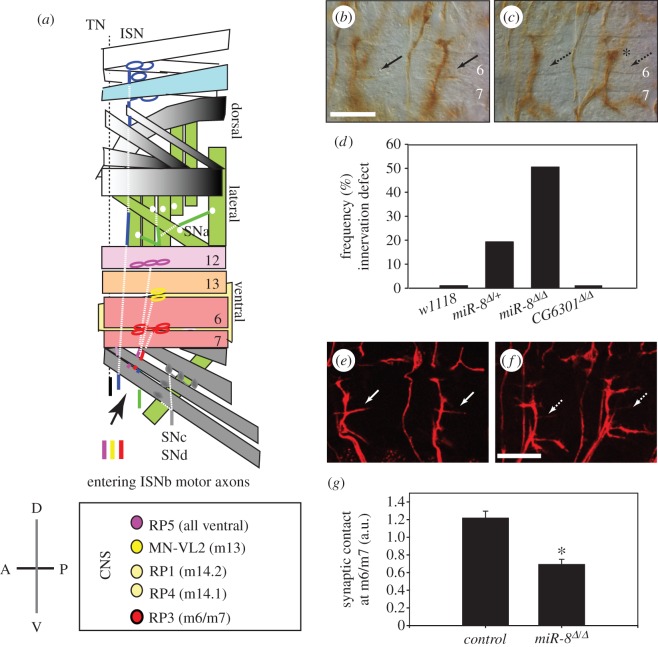
miR-8 promotes embryonic motor axon ISNb innervation along muscle 6 and 7 cleft. (*a*) Schematic of the neuromuscular connectivity. The axons of motor neurons (coloured circles) exit neuropile in the central nervous system (CNS) along three major nerve trunks: ISN, TN and SN which branch out further to innervate dorsal, lateral and ventral muscle fields. Muscles are colour-matched with the representative examples of innervating motor neuron partners. ISNb branch (highlighted in bold), which consists of axons from motor neurons with distinct dendritic morphology, stereotypic orientation and position in relation to the anterior commissures (AC), posterior commissures (PC) and longitudinal connectives in the ventral nerve chord, defasciculates from the ISN root to innervate ventral longitudinal (pink shades) and oblique (yellow shades) muscles 6, 7, 12, 13, 14.1 and 14.2. RP3 motor neuron (highlighted in bold) specifically innervates muscles 6 and 7 (m6/m7) to form synapses. (*b*,*c*) Motor axon ISNb termini and innervation along m6/m7 cleft in stage 17 wild-type and *miR-8^Δ/Δ^* mutant embryos by anti-FasII immuno­staining. Scale bar = 10 μm. (*b*) Normal motor axon ISNb branching pattern and specific axon innervation along m6/m7 cleft (solid arrows) in isogenic *w^1118^* embryos. (*c*) Motor axon ISNb branching pattern with weak innervation along m6/m7 cleft in *miR-8^Δ/Δ^* mutant embryos (broken arrows). Weak innervation of m6/m7 by the ISNb branch is characterized by the complete absence of anti-FasII immunoreactivity *in situ* in the most severe cases or otherwise by substantially reduced length of axon innervation along m6/m7 cleft. The asterisk indicates the m13/m30 cleft with increased accumulation of FasII. (*d*) Quantification of the frequency of ISNb innervation defect at m6/m7 cleft in wild-type and mutant embryos with genotypes as described in the bar graph. The frequency of defective innervation is expressed as a percentage of affected hemisegments (*n* = 180 for isogenic *w1118*; *n* = 114 for *miR-8^Δ/+^*, *n* = 285 for *miR-8^Δ/Δ^*, *n* = 164 for *CG6301^Δ/Δ^*, *p* = 0.76 × 10^–4^, one-way ANOVA). (*e,f*) ISNb axon termini and innervation along m6/m7 cleft in control stage 17 *islet-*τ*-mycGFP/+* (*e*; solid arrows) and in *miR-8^Δ/Δ^; islet-*τ*-mycGFP/+* mutant embryos (*f*; broken arrows) by anti-GFP immunostaining. Scale bar = 10 μm. (*g*) Quantification of the reduced synaptic coverage along m6/m7 cleft in *islet-*τ*-mycGFP/+* control and *miR-8^Δ/Δ^;islet-*τ*-mycGFP/+* mutant embryos. Synaptic coverage is represented by integration of GFP immunofluorescence intensity along the m6/m7 cleft normalized to the signal intensity along the m12/m13 cleft, which is unaltered by miR-8 deletion and serves as the internal control (*n* = 24; *p = 5.48 × 10^–6^, Student's *t*-test).

We uncovered the miR-8 locus in a genetic screen for Abl tyrosine kinase modifier genes (C. S. Lu & D. Van Vactor 2009, unpublished data) and showed that miR-8 promotes late larval expansion of the NMJ via postsynaptic repression of the actin-binding protein Enabled (Ena; [23]). However, the early onset of miR-8 expression raised the question of whether this miRNA might influence the initial stages of NMJ development. The profile of miR-8 expression was previously characterized by Northern blots of the major life cycle stages [37–39], indicating that miR-8 expression begins during embryonic stages. We confirmed this result using a more sensitive quantitative RT-PCR assay with greater temporal resolution (see the electronic supplementary material) and discovered a major peak of miR-8 at 10.5–13 h AEL as well as a minor peak at 20–22 h AEL (electronic supplementary material, figure S1). Synaptogenesis at the *Drosophila* NMJ starts at approximately 13 h AEL after motor axon growth cones have contacted body wall muscles and expanded filopodia to explore these [40,41]. During the next 2 h of development, exuberant axonal arborizations over non-target muscles are normally withdrawn and the exploratory membrane interfaces become restricted to specific synaptic sites (target refinement stage at 13–15 h AEL; figure 1*a*). From 14 h AEL onwards, postsynaptic specializations gradually accumulate glutamate receptors while synaptic vesicles accumulate at nascent presynaptic active zones and individual synaptic boutons appear [42,43].

A major peak of miR-8 expression conspicuously coincides with the refinement of motor axon contacts with specific target muscles. This prompted us to examine embryonic NMJ morphology in mutants lacking miR-8. One of the best-characterized groups of synapses in this system is a domain of innervation formed by the intersegmental nerve branch b (ISNb, a group of seven motor axons) on the ventral longitudinal and oblique muscles (m6, m7, m13, m12 and m14.1, m14.2, respectively; figure 1*a,b*). In *wild-type* embryos, as in embryos homozygous for a *miR-8* null mutation generated by targeted deletion (see the electronic supplementary material, figure S2), ISNb axons showed normal trajectories to reach their ventral muscle target domain. However, subsequent to the target recognition stage, we discovered an innervation defect in the *miR-8* null mutants. Using the IgCAM Fasciclin II (FasII) as a marker for embryonic motor axons [44], we found that the innervation of the cleft between m6 and m7 was undetectable or the length substantially reduced in *miR-8* null embryos. To compare expressivity of defective ISNb innervation at m6/m7, the percentage of segments in which the anti-FasII staining was less than half the length typical of wild-type NMJs was quantified blind of genotype. In all *miR-8* mutants examined, approximately 50% of all A2–A7 hemisegments displayed this defect (figure 1*c*,*d*). The reduction of FasII staining at the m6/m7 muscle cleft was often accompanied by an increase in staining at the more distal m13 cleft. To confirm that this phenotype was miR-8 specific, we compared miR-8 allelic combinations with controls of near identical genetic background where, instead of miR-8, the adjacent protein-coding gene (CG6301) had been deleted (electronic supplementary material, figure S2). While these control homozygous CG6301Δ embryos showed no ISNb phenotype, we found that even removal of one copy of miR-8 was sufficient to induce an ISNb defect at an intermediate frequency (approx. 20%; figure 1*d*), suggesting a dose-dependent relationship between miR-8 and ISNb development. Because FasII localization to distinct regions of the motor axon is regulated in the central nervous system (CNS) [45–47], we also compared ISNb terminal morphology in *miR-8* nulls and isogenic controls using an independent marker system (islet-GFP: tau-myc-EGFP under control of *islet* regulatory regions [34]; figure 1*e*,*f*). The islet-GFP marker revealed some degree of innervation at most m6/m7 targets in *miR-8* mutants. However, the synaptic contact as assessed by integration of GFP intensity along the m6/m7 muscle cleft is reduced by nearly 40% compared with controls (figure 1*g*). This analysis showed that although FasII localization at the m6/m7 synaptic site was more severely affected than the elaboration of motor axon terminals, miR-8 is required from the earliest stage of synapse formation, consistent with the early miR-8 expression peak.

### (b) Synaptic target recognition between RP3 motor axon terminals and target muscles 6 and 7 is affected by miR-8

**Figure 2.**
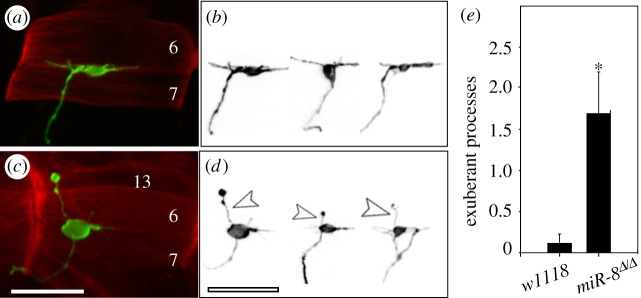
Synaptic target refinement is dependent on miR-8. (*a*) RP3 motor axon terminal (green) in a late stage 16 *islet-*τ*-mycGFP/+* control embryo at its target, extending along the cleft between m6/m7 (red). RP3 motor neurons and axon terminals were labelled by anterograde DiI injection and muscles were counterstained with Alexa Fluor 647 phalloidin. (*b*) Tracings of additional RP3 axon terminals in control embryos. (*c*) RP3 motor axon ISNb terminals reach muscle targets but are not confined to the m6/m7 cleft in late stage 16 *miR-8^Δ/Δ^* mutant embryos. The example here shows an RP3 motor axon process overextends to non-target muscle m13, which is never observed in wild-type and control embryos at this stage. (*d*) Tracings of RP3 motor axon terminals in *miR-8^Δ/Δ^;islet-*τ*-mycGFP/+* mutant embryos that have exuberant sprouting of axonal processes (open arrowheads). Scale bar = 10 μm. (*e*) Quantification of exuberant processes. An exuberant process is characterized as a sprouting within the RP3 motor axon termini measured to be 10% or longer than the average length of the main branch. The number of sproutings over a defined length of axon is used for comparison between control and mutant embryos (*n* = 12; *p = 2.92 × 10^–3^, Student's *t*-test).

One useful feature of the *Drosophila* system is the extent to which the identities of the motor neurons that make individual NMJs have been defined. Motor neurons RP3 and RP5 innervate the ventral muscles m6/m7 and of these RP3 is the first neuron to form a functional synapse at this target. In order to determine whether the defects in *miR-8* mutant ISNb morphology observed with FasII and islet-GFP represent a failure to assign RP3 cell fate or an early defect in axon guidance, we performed anterograde DiI injections. At 15 h AEL, RP3 motor neurons of wild-type and *miR-8* null mutant embryos showed normal morphology of somata and dendritic arbors (not shown) and their axon terminals successfully reached the m6/m7 cleft in all cases (figure 2*a*,*b*; *n* = 12 cases for wild-type and *miR-8^Δ^/miR-8^Δ^*). However, in 15 h-old *miR-8* mutants RP3 axon terminals did show two abnormalities: first, we noted a fourfold increase in exuberant sprouting of filopodia and less well-defined elaboration of the NMJ between m6/m7 (figure 2*d*,*e*); second, in several instances RP3 axon terminals extended to a neighbouring non-target muscle, m13, and formed varicosities on m13 (arrowheads in figure 2*d*; *n* = 3 of 12). These observations are consistent with both the decreased FasII staining at the m6/m7 cleft and the increase in FasII labelling we found at m13 in *miR-8* null embryos (asterisk, figure 1*c*). Consistent with our FasII and islet-GFP data, approximately 50% of the RP3 motor axons manifested either reduced target innervation area and/or increased exuberant sprouting of filopodia. These results confirmed that loss of miR-8 had little effect on RP3 specification or axon pathfinding into the correct target domain. Rather, the failure of *miR-8* mutants to restrict exploratory membrane contacts and consolidate innervation at the m6/m7 synaptic site suggested a role for miR-8 during the target refinement stage.

### (c) A proteomic screen for miR-8 effectors *in vivo* identifies functional clusters for synapse development

Understanding the cellular mechanism(s) by which miR-8 promotes accurate innervation of m6/m7 required the identification of relevant downstream effector genes. Our previous *in silico* (using TargetScan Fly 5.1) and expression analysis of mRNAs to determine candidates that might be directly regulated by miR-8, identified the actin-associated protein Ena as a key effector that accounts for much of miR-8 NMJ function at the larval stage [23]. However, to our surprise, both over-expression assays and double-mutant genetic rescue assays revealed that Ena cannot account for miR-8 function during NMJ formation in the embryo (electronic supplementary material, figure S3).

**Figure 3.**
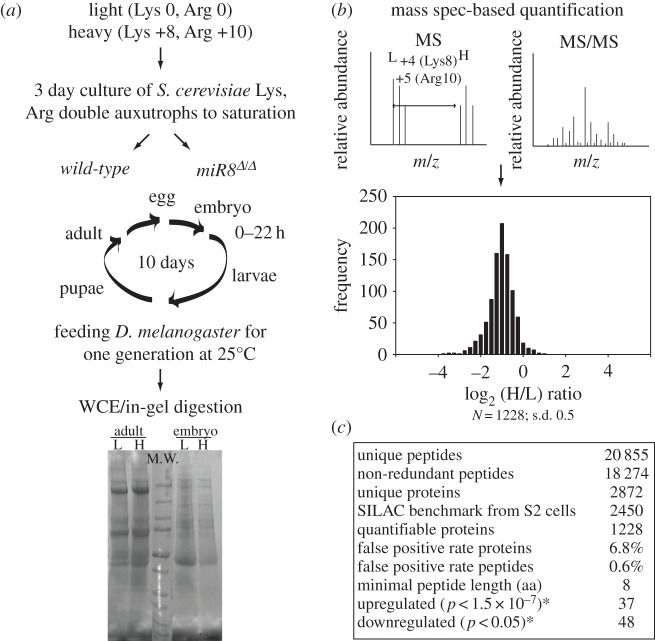
Profiling *in vivo* gene effectors downstream of miR-8 by comparative proteomics. (*a*) Schematic work flow for the metabolic labelling of essential amino acids lysine and arginine for all proteins in intact *Drosophila* (‘*in vivo* SILAC’). ‘Light’ ^12^C_6_^14^N_2_ l-lysine and l-arginine or ‘heavy’ ^13^C_6_^15^N_2_ l-lysine and l-arginine with a predicted mass shift in daltons from Lys (0), Arg (0) to Lys (+8) and Arg(+10) were added to the drop-out media to grow a Lys, Arg double auxotroph *S. cerevisiae* strain to saturation. Aliquots of yeast culture were fed as the food source to wild-type isogenic *w^1118^* and *miR-8^Δ/Δ^* mutant embryos from 10 h AEL on for one generation at 25°C. Proteins from whole cell extracts (WCE) of native fly tissues were resolved by SDS-PAGE and digested by trypsin to release peptide mixtures containing labelled Lys and Arg in the C-terminus for further quantification. (*b*) Quantification of peptide abundance and relative ratio of heavy (H: *miR-8^Δ/Δ^*) to light (L: *w^1118^*) by liquid chromatography and tandem mass spectrometry (LC-MS/MS). In the top panel, the illustration depicts unambiguous separation of peptide clusters on MS spectra for proteins labelled with heavy Lys and Arg and the light isotopes thanks to a sizable shift in relative molecular mass. Abundance is measured by peak amplitudes of the labelled and label-free peptides on MS spectra. Peptide identification is conducted from pattern recognition searches between composite peptide reference MS/MS databases and experimental MS/MS spectra. In the bottom panel, the distribution of quantifiable proteins are plotted as a histogram of log_2_-fold changes in H/L ratio indicating the relative expression levels of proteins found in the labelled *miR-8^Δ/Δ^* mutant and the*^1118^* embryos. (*c*) Summary table of key statistics for the comparative proteomic profiling using SILAC fly embryos.

In order to define a more complete set of candidate downstream effectors, we turned to a quantitative mass spectrometry-based approach using an adaptation of SILAC (Stable Isotope Labeling with Amino acids in Cell culture) for use in whole animals [48–50]. We surveyed and compared the proteomes directly from the wild-type and *miR-8* null embryos differentially labelled with ^13^C and ^15^N on the Lys and Arg residues (figure 3*a*). ^13^C-Lys/Arg provided unequivocal differentiation between labelled peptides derived from the same proteins but isolated from two different genetic backgrounds (figure 3*b*, top panel). This differential labelling workflow included automatic quantification of the peptide mixture prior to the identification of fragmented peptides to confirm that 98.5% of heavy ^13^C-Lys/Arg had already been incorporated in F_1_ generation adults (see §2*d*). We also found negligible contribution of Arg to Pro conversion to the accuracy of quantification. Quantifiable proteins in the *miR-8* null and wild-type distribute in a bell-shaped curve fitted to a normal distribution along the log_2_ axis for the heavy (*miR-8* null) relative to light (*wild-type*) ratios (figure 3*b*, bottom panel). Approximately 95% of all quantifiable proteins cluster around the population mean and hence we applied 2 s.d. as the cutoff threshold to catalogue proteins with the most substantial changes. We found 37 proteins with upregulation more than 180% and 48 proteins that were downregulated more than 55% in the absence of miR-8 (figure 3*c*).

To prioritize miR-8-dependent proteins that might contribute to the *miR-8* null embryonic phenotype, we analysed the list of proteins with the highest differential expression ratios from *in vivo* SILAC using a publically available DAVID functional pathway analysis and ontogeny tool (electronic supplementary material). In contrast to the microarray profiling of potential targets of miR-8 which revealed diverse functional classes with little class-specific enrichment except for the ribosomal and translation process [26], this proteomic strategy identified other functional clusters enriched significantly above the background proteome based on one-tailed Fisher exact probability of over-representation. Interestingly, the top 10 most significant functional clusters of proteins with altered expression in the *miR-8* mutant embryo, as compared to the background proteome, included ‘synapse organization and NMJ development’ (electronic supplementary material, table S1).

### (d) miR-8 is required for immunoglobulin superfamily cell adhesion molecules Fasciclin III and Neuroglian to localize to synaptic regions

Based on our characterization of *miR-8* mutant defects in synaptic innervation, we decided to investigate further how miR-8 effects local synaptic adhesion. Within this class of miR-8-dependent candidates, seven out of nine show various neuroanatomy defective phenotypes when mutated and are normally expressed in embryonic neurons and/or muscles based on published literature and transcriptome analysis performed by the *Drosophila* model organism Encyclopedia of DNA Elements (modENCODE) project [51]. However, these miR-8-dependent candidate effectors are not involved in initiation of de novo synaptogenesis and lack seed sequence homology to be direct targets of miR-8 (see the electronic supplementary material and §2*d*). The fact that their levels decrease in the *miR-8* null background (electronic supplementary material, table S2) is consistent with miR-8 playing a role in stabilizing target recognition during synapse development.

**Figure 4.**
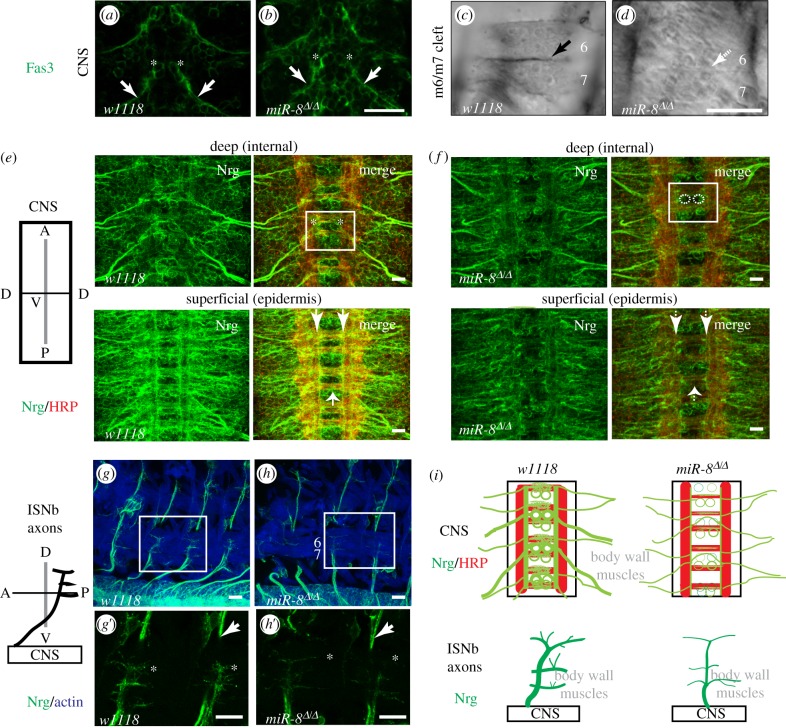
miR-8 affects *in situ* expression of IgCAMs FasIII and Nrg in primordial synapses. (*a*–*d*) Anti-FasIII immunohistochemistry in the CNS and muscles m6/m7 cleft of stage 16 embryos. FasIII is expressed in RP3 motor neurons in the neuropile (asterisks) and their axons (arrows) in both (*a*) wild-type *w^1118^* and (B) *miR-8^Δ/Δ^* mutant. (*c*) FasIII immunostaining along m6/m7 cleft is present in wild-type at this stage (solid arrow). (*d*) Reduced FasIII immunostaining along m6/m7 cleft (broken arrow) in *miR-8^Δ/Δ^* mutant. Scale bar = 10 μm. (*e*–*h*) Anti-Nrg immunohistochemistry (green) in the CNS and ISNb motor axon targeting domain in early stage 16 embryos. Anti-HRP (red) counterstains the neuronal cell bodies and processes in the CNS (*e*,*f*) and phalloidin counterstains muscle actin (blue) in (*g*,*h*). (*e*) CNS expression of Nrg in motor neurons, including RP3s in the box region (asterisks) is visible in the most internal focal planes of the ventral nerve chord, and in longitudinal and commissural axon tracts (arrows) of wild-type *w^1118^* embryos. The Nrg isoform expressed in the cell membrane of epidermal cells is visible in the superficial focal planes. (*f*) Decreased Nrg immunostaining signal in the CNS of *miR-8^Δ/Δ^* mutant embryos. Outlines of RP3s inside the box region are highlighted with dotted lines and axon tracks indicated by arrows. (*g*) Accumulation of Nrg immunostaining in the ISNb motor axon (green) innervations in the target ventral muscles domain (blue) in wild-type *w^1118^* embryos. (*g*’) View of the boxed region in (*g*) in greater detail. Filopodial tips of ISNb growth cones (asterisks) and peripheral axons (arrows) are indicated. (*h*) Decreased Nrg accumulation in the ISNb motor axon (green) in *miR-8^Δ/Δ^* mutant embryos. Growth cones (asterisks) and peripheral axons (arrows) in *miR-8^Δ/Δ^* mutant embryos are indicated. (*h*’) View of the boxed region in (*h*) with higher magnification. Scale bar = 10 μm. (*i*) Summary diagram of Nrg expression in the CNS and ISNb axons.

Among synaptic IgCAMs identified in our SILAC dataset, two of them had been previously implicated in ISNb development: FasIII [52,53] and Nrg [32,54]. In *wild-type* embryos, FasIII is coordinately expressed on both the RP3 motor axon and at the specific central region of the m6/m7 muscle cleft where RP3 will form its synaptic terminal [55]. FasIII accumulates at the synaptic target site on m6/m7 even when motor innervation is absent [56], suggesting that this IgCAM accumulates due to homophillic contact on abutting m6 and m7 membranes and thus presages the site of synaptic contact. Although FasIII in *miR-8* null showed a 58% reduction relative to in *wild-type* in our SILAC dataset, this could reflect an underestimate at synapses because FasIII expression in the epidermis accounts for a major source [35], and these experiments were performed with whole embryo lysates that cannot distinguish between different sites of protein expression. Thus, we examined the distribution of FasIII with *in situ* immunocytochemistry. Consistent with the SILAC result, we found a 34–65% decrease of anti-FasIII signal intensity in the dorsal epidermal stripes of *miR-8* null embryos (data not shown). By contrast, RP3 motor axon expression of FasIII in *miR-8* nulls was indistinguishable from controls (figure 4*a*,*b*; *n* = 10), thus confirming the normal cell fate and axon pathfinding of RP3 in these mutant embryos. The morphology and position of the ventral muscles in *miR-8* mutants was also indistinguishable from *wild-type*. However, when we examined FasIII accumulation on the adjoining surfaces of m6 and m7, it was absent or barely detectable in 67% of hemisegments of all *miR-8* mutant embryos examined (*n* = 8, figure 4*c*,*d*). This striking change of FasIII expression at m6/m7 in *miR-8* mutants validated postsynaptic FasIII as a factor downstream of miR-8, and suggested that miR-8 is required to define the synaptic site to which RP3 growth cones are attracted during motor axon targeting.

Based on the coordinated pre- and postsynaptic pattern of FasIII expression at the m6/m7 embryonic NMJ, Chiba and colleagues proposed that FasIII directs RP3 target selection, based on evidence of altered RP3 targeting upon misexpression of FasIII on non-target ventral muscles [52]. However, FasIII loss of function alone did not change the site of RP3 innervation [52], suggesting that additional cell-surface proteins contribute to precise target recognition at the m6/m7 cleft. In this regard, Nrg was a promising candidate due to its expression on ISNb motor growth cones and ISNb axon phenotypes observed in *nrg* mutants [32]. Nrg is the *Drosophila* orthologue of the neural IgCAM L1, the causal factor for multiple neurological defects associated with CRASH syndrome patients (Corpus callosum hypoplasia, Retardation, Adducted thumbs, Spasticity and Hydrocephalus) [57]. The overall expression of Nrg was decreased to 58% in *miR-8* null embryos when compared with *wild-type* controls in our SILAC dataset (electronic supplementary material, table S2). Two distinct *Drosophila* Nrg isoforms are expressed in embryo: Nrg^167^ is ubiquitous, while Nrg^180^ is neuronal-specific [58]. We characterized spatial changes in Nrg^180^ by *in situ* by immunocytochemistry in wild-type and *miR-8* null embryos. In the ventral nerve cord, loss of miR-8 leads to a reproducible decrease in anti-Nrg signal in the longitudinal connectives, anterior and posterior commissures, and in multiple neurons including RP3 located in the neuropile, as compared to *wild-type* controls (arrows and box insert in figure 4*e*,*f*; *i* (top panel); *n* = 12; see §2*b*). In the periphery, Nrg normally accumulates along peripheral nerves (figure 4*g* and arrow in figure 4*g*’) and on the filopodia of wild-type ISNb motor growth cones as they explore the ventral muscle field (asterisks in figure 4*g*’). However, in *miR-8* null mutant embryos, Nrg levels on these ISNb growth cones are substantially decreased (*n* = 7, figure 4*h* and asterisks in figure 4*h*’) and 72% of hemi-segments analysed showed at least a 30% reduction. This decrease in Nrg levels on motor axon growth cones as they explore their target area occurs locally, as intersegmental axons on their trajectory towards dorsal muscle targets showed levels of Nrg that were indistinguishable from controls (see arrows in figure 4*g*’,*h*’). These observations confirmed that normal expression and localization of neuronal Nrg require miR-8.

### (e) Presynaptic Neuroglian acts downstream of miR-8 and genetically interacts with Fasciclin III

**Figure 5.**
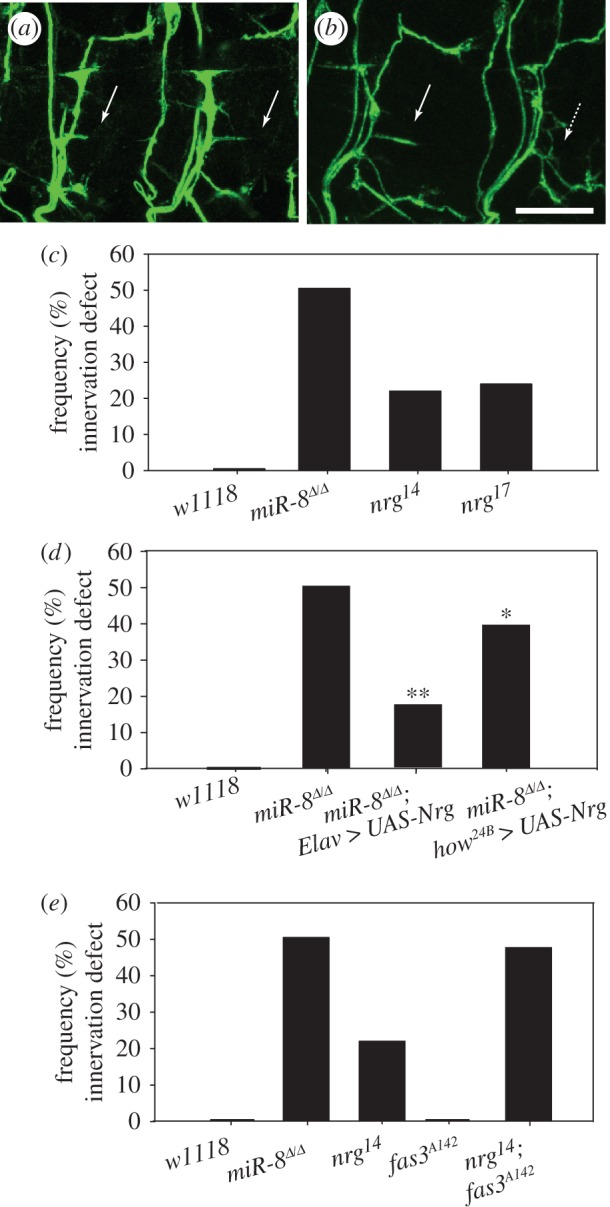
Trans-synaptic coordination of Nrg with FasIII downstream of miR-8 is essential for robust ISNb motor axon innervations at m6/m7. (*a*) Normal ISNb branching pattern and specific innervation along m6/m7 cleft (solid arrows) as revealed by anti-FasII immunostaining in stage 17 wild-type *w^1118^* embryos. (*b*) Weak ISNb innervations similar to those observed in *miR-8^Δ/Δ^* mutant embryos (broken arrow) and normal innervations (solid arrow) along m6/m7 cleft in the adjacent hemisegments of a loss-of-function *nrg^14^* mutant embryo. Scale bar = 10 μm. (*c*) Quantification of the frequency of ISNb innervation defect along m6/m7 cleft in wild-type, *miR-8^Δ/Δ^*, and *nrg* mutant embryos (*n* = 180 for *w^1118^*; *n* = 285 for *miR-8^Δ/Δ^*; *n* = 136 for *nrg^14^/nrg^14^*; *n* = 100 for *nrg^17^/nrg^17^*). The homozygous or hemizygous *nrg^14^* and *nrg^17^* are not significantly different from each other (*p* = 0.172, Student's *t*-test). (*d*) Quantification of the pre- and postsynaptic rescue by full-length Nrg transgene *UAS-Nrg^180^* in the *miR-8^Δ/Δ^* background (*n* = 180 for isogenic *w^1118^*; *n* = 285 for *miR-8^Δ/Δ^*; *n* = 149 for *miR-8^Δ/Δ^;Elav* > UAS-Nrg, **p = 1.42 × 10^–8^, Students *t*-test; *n* = 96 for *miR-8^Δ/Δ^;how^24B^* > *UAS-Nrg*; *p = 0.003, Student's *t*-test). (*e*) Quantification of the genetic interaction between Nrg (*nrg*^14^) and FasIII (*fas3^A142^*). *n* = 180 for *w^1118^*; *n* = 285 for *miR-8^Δ/Δ^*; *n* = 136 for *nrg^14^/nrg^14^*; *n* = 120 for *fas3^A142^*; *n* = 118 for *nrg^14^/nrg^14^;fas3^A142^/fas3^A142^*. The percentage of weak m6/m7 innervation in the *nrg;fas3* double mutant is comparable to that of *miR-8^Δ/Δ^* mutant embryos (*p* = 0.384, Student's *t*-test).

Like its human counterpart L1-CAM [59], Nrg is required for the accurate connectivity of multiple axons in *Drosophila*. In the adult fly, loss or mutation of Nrg protein leads to reduced numbers of axonal terminals forming synapses in visual and escape reflex circuits [60,61]. Nrg is also essential for maintaining stable synaptic architecture at larval NMJs [62]. However, in embryos, Nrg has been shown to support ISNb motor axon guidance and targeting [32]. Next, we wanted to determine the functional contribution of the miR-8 downstream effectors, Nrg and FasIII, to the formation of NMJs in the embryo. Using anti-FasII staining of stage 17 motor axons, we applied the same parameters as described for figure 1*d* and quantified the frequency of diminished or absent innervations at the m6/m7 cleft. We found that two different *nrg* alleles (*nrg^14^* and *nrg^17^*) display a synaptic defect highly reminiscent of that observed in the *miR-8* null, though with reduced penetrance (figure 5*b*,*c*). If lower levels of Nrg on ISNb growth cones were responsible for the *miR-8* NMJ phenotype, we reasoned that elevation of Nrg (with *UAS-nrg*) in embryos lacking miR-8 should compensate and restore innervation of the m6/m7 cleft. While neural-specific elevation of Nrg expression using an *Elav-GAL4* driver did not generate any ISNb defect on its own (not shown), it restored 66.7% of weak synaptic contacts in a *miR-8* null background (figure 5*d*), thus supporting a model where miR-8 promotes ISNb NMJ formation at m6/m7 by maintaining levels of Nrg in these motor axon growth cones as they explore their target territory. To confirm the presynaptic specificity of Nrg function, we also examined the impact of Nrg over-expression on the target muscle cells (using *how^24B^-GAL4*). In contrast to neuronal expression, elevation of Nrg in muscles induced a de novo ISNb axon arrest phenotype (not shown). Postsynaptic expression of *UAS-nrg* in the *miR-8* null suppressed only 10% of the innervation defect at m6/m7 in the sub-population of motor axons that reach the target domain (figure 5*d*). Thus, we concluded that mainly changes in presynaptic Nrg expression contribute to the ISNb phenotype observed in *miR-8* null mutant embryos.

While NMJ formation at the m6/m7 synaptic site requires Nrg, the fact that strong *nrg* alleles display roughly half the penetrance of *miR-8* nulls for this phenotype suggested that some additional effector(s) were involved. Given the striking change in synaptic FasIII accumulation in *miR-8* mutants (figure 4*d*), we wondered whether the combined influence of Nrg and FasIII might explain the higher penetrance of the *miR-8* mutant phenotype, even though elimination of FasIII alone is not sufficient to induce the defect. To test this possibility, we genetically removed both FasIII and Nrg at the same time and then quantified the m6/m7 innervation using anti-FasII staining. Interestingly, introduction of a FasIII null allele (*fasIII^A142^*, which has no ISNb phenotype alone) into an *nrg^14^/nrg^14^* mutant background more than doubled the frequency of m6/m7 innervation defects, as compared to embryos singly mutant for *nrg^14^* (figure 5*e*). The fact that the m6/m7 innervation phenotype in *nrg^14^/nrg^14^;fasIII^A142^/fasIII^A142^* double-mutant embryos matches the strength and penetrance of the defects found in *miR-8* homozygous nulls is consistent with a model where a combination of pre- and postsynaptic IgCAMs are key downstream effectors of miR-8 for NMJ formation.

## 4. Discussion

Although miRNAs hold substantial promise as regulators of synapse development, maintenance and plasticity, very little is known about the roles of particular miRNA genes in matching axon terminals with appropriate synaptic partners in the embryonic nervous system. Our findings identify a novel role for miR-8 during the refinement of initial synaptic contacts in the *Drosophila* embryo. Through a combination of comparative quantitative proteomics and developmental genetic analysis, we find that miR-8-dependent expression of the synaptic CAMs Nrg and FasIII can account for the abnormal behaviour of RP3 motor neuron synaptic terminals in *miR-8* mutants. Unlike late larval stages where only we find evidence for postsynaptic miR-8 control of NMJ morphogenesis [23], presynaptic sequestration of embryonic miR-8 moderately increases the frequency of innervation defects of ISNb axon along m6/m7 (electronic supplementary material, figure S4) and is required for normal localization of Nrg on ISNb motor growth cones. Since embryonic miR-8 also is required for deployment of FasIII in the specific region of m6/m7 cleft normally innervated by ISNb, we propose that miR-8 acts to coordinate synaptic CAMs on both sides of the synapse.

The targeting of motor axons to their respective muscle partners in *Drosophila* has been mapped at single cell resolution, revealing a remarkably specific and stereotyped pattern of innervation. To provide sufficient information content for robust and specific target recognition, popular models often rely on a combinatorial code of many cell-surface proteins, including IgCAMs, leucine-rich repeat adhesion molecules (LRRs) and receptors for diffusible cues (e.g. Wnt, Netrin, Semaphorins). However, such models have proved difficult to validate *in vivo*. Previous experiments with the diffusible Semaphorin II (Sema II) and Netrin during RP3 innervation of m6/m7 did suggest a combinatoral mechanism [63], but functional synergy between these secreted factors was only observed via Sema II misexpression. While combinatorial target specification had not been previously tested for synaptic CAMs in *Drosophila*, our current data demonstrate combinatorial synergy between Nrg and FasIII at the m6/m7 NMJ via loss of endogenous gene function. During this stage, miR-8 appears to play a rather subtle role in refining the target recognition of motor axon terminals at the m6/m7 cleft by regulating the spatial distribution of Nrg and FasIII. While additional experiments will be necessary to prove that miR-8 function at the m6/m7 NMJ can be fully accounted for by Nrg and FasIII, the nature of this early phenotype suggests that miR-8 contributes to the accuracy or robustness of motor connectivity, consistent with the roles of many miRNAs in fine-tuning of genetic circuits [64].

It is thought that a set of neuronal and muscle transcription factors determines the deployment of genes required to achieve accurate connectivity in the neuromuscular system [65], although the precise relationship between the targeting receptors and the upstream factors that control their expression is just beginning to emerge. For example, the transcription factor Tey has been proposed as a targeting factor in m12 via repression of the repellent cell-surface protein Toll [66]. Because neither the Nrg nor FasIII gene contains sites with homology to the miR-8 seed sequence complement, and because the levels of these synaptic IgCAMs are decreased in *miR-8* mutants, we believe that miR-8 controls Nrg and FasIII via some intermediate regulatory component(s). While the transcription factors upstream of FasIII have yet to be defined, genetic studies suggest that neuronal Nrg expression falls under the negative regulation of the homeobox protein Engrailed (En, [67]). When En is overexpressed in all post-mitotic neurons, Nrg immunostaining in the embryonic CNS, sensory and motor axon pathways including RP3 all decreased [67]. In addition, the 3′-UTR of the *en* mRNA contains one seed sequence homology site for miR-8 that is well conserved across *Drosophilid* species (not shown). Whether En serves as an intermediate between miR-8 and functional effector proteins in the embryonic nervous system will require careful quantitative analysis of En expression in *miR-8* mutants, plus additional *in vitro* and *in vivo* functional validation.

Recent work has begun to suggest roles for miRNA function in axon growth and guidance [68–70] in addition to a larger body of work on miRNA regulation of dendritic development and synapse plasticity [12]. However, little is known about miRNA control of the initial formation and specificity of synaptic connectivity. Our studies of miR-8 and two downstream synaptic IgCAMs suggest that miRNA can coordinately regulate pre- and postsynaptic effector molecules. Our data also indicate that Nrg and FasIII act synergistically to ensure robust synaptogenesis *in vivo*, providing evidence for combinatorial specification of synaptic connectivity.

## Supplementary Material

Supplementary Materials and Methods; Figures and Tables
