# Supplementary material for: MicroRNA-8 promotes robust motor axon targeting by coordinate regulation of cell adhesion molecules during synapse development
Source: Philos Trans R Soc Lond B Biol Sci. 2014 Sep 26;369(1652):20130517. doi: 10.1098/rstb.2013.0517 (PMC4142038; doi:10.1098/rstb.2013.0517)
Supplement: Supplementary Materials and Methods; Figures and Tables [file rstb20130517supp1.pdf]

Phil. Trans. R. Soc. B. **369** doi: 10.1098/rstb.2013.0517

Supplemental Information

MicroRNA-8 Promotes Robust Motor Axon Targeting By Coordinate Regulation of Cell Adhesion Molecules during Synapse Development

Cecilia S. Lu[1, 2, 3], Bo Zhai[1], Alex Mauss[4, 5], Matthias Landgraf[4], Steven Gygi[1] and David Van Vactor[1, 2, 3, 6]

[1] Department of Cell Biology and [2] Program in Neuroscience, Harvard Medical School, Boston, MA 02115

[3] Okinawa Institute of Science and Technology Graduate University

[4] Department of Zoology, University of Cambridge, Cambridge, UK

[5] Max Planck Institute of Neurobiology, Martinsried, Germany

[6] Corresponding author email: davie_vanvactor@hms.harvard.edu

© 2014 The Authors under the terms of the Creative Commons Attribution License http://creativecommons.org/licenses/by/3.0/, which permits unrestricted use, provided the original author and source are credited.

Materials and Methods:

Quantitative RT-PCR analysis

Whole *Drosophila* embryos from synchronized egg-lays were collected every two hours, aged at 25°C according to developmental time points (0-3 hr, 3-7.5 hr, 7.5-10 hr, etc.) and a small batch of each time point removed for examination of staging based on gut morphology (1) before they were dechorionated in 50% bleach for 2 min and snap frozen in liquid nitrogen. Total RNA was extracted and purified from these embryos using mirVana miRNA isolation kit (Ambion), polyadenylated with poly A polymerase (Invitrogen), and reverse transcribed using a mixture of a Universal RT Primer (Invitrogen), DTT, dNTPs, glycerol and Superscript III reverse transcriptase into cDNA following standard protocols. Amplification of miR-8, miR-8S (anti-sense control) and 18S ribosomal RNA (internal standard) transcripts from different developmental time points was carried out by PCR for 27, 29, 31, 33, 35 cycles in a thermocycler (MJ Research PTC-200) following manufacturer's protocols. The PCR products at cycle 29 were removed for electrophoresis and further quantification by ImageQuant on Fuji LAS 3000. The quantitative PCR reaction mix consists of 18S rRNA primers and its Competimer (Ambion) as internal control, miR-8 and miR-8S specific primers (Integrated DNA Technologies) and qPCR SuperMix from NCode Express SYBR GreenER miRNA qRT-PCR kit (Invitrogen).

Primers for two-sided and genomic PCR analysis of miR-8 $^{\Delta/\Delta}$ mutant

d01682-fwd: 5'-CGTCACCCAGTCGTTTTT-3'
XP(-)-rev: 5' TACTATTCCTTTCACTCGCACTTATTG-3'

WH(+)-fwd: 5'-CCTCGATATACAGACCGATAAAAC-3'
f05125-rev: 5'-AATGGTAGCAGGCTTCAAATACAACT-3'

dMiR-8 fwd: 5'-GCGCACTCGAGCTAATTAGCAAGGACATCTG -3'
dMiR-8 rev: 5'-AATGGTAGCAGGCTTCAAATACAACT-3'

RNase protection assay

Total RNA was extracted from 0-22h old *Drosophila* embryos using acidic phenol and chloroform with chaotropic salts and the small RNAs (<200 nt) fraction enriched using differential ethanol precipitation followed by glass-fiber filtration (Ambion). 6000 Ci/mmole of radioactive labeled anti-sense miR-8 and control probes were prepared from HPLC-purified RNA oligonucleotides (Integrated DNA Technologies) followed by phosphate transfer from [γ-^{32}P] ATP (Perkin-Elmer) by T4 polynucleotide kinase reaction (Ambion). The sample RNAs were hybridized with probes at 42°C over night, subjected to RNase A/T1 digestion (Ambion) and resolved by electrophoresis on a denaturing 15% polyacylamide/8M urea gel. The protected RNA fragments were detected by autoradiography.

miR-8 probe: 5'-ACAUCUUUACCUGACAGUAUUAUUCUUA-3'
control let-7 probe: 5'-ACUAUACAACCUACUACCUCAAAAUUCUUA-3'
control miR-16 probe: 5'-CGCCAAUAUUUACGUGCUGCUACCAGAG-3'

LC−MS/MS analysis

Peptides mixture were loaded by a Famos autosampler (LC Packings, San Francisco, CA) onto a 125 μm (i.d.) × 18 cm fused silica microcapillary column in-house packed with C_{18} reverse-phase resin (Magic C18AQ; 5-μm particles; 200-Å pore size; Michrom Bioresources, Auburn, CA), and separated with an Agilent 1100 series binary pump with in-line flow splitter across a 60-min linear gradient ranging from 6% to 28% acetonitrile in 0.125% formic acid. The LTQ FT hybrid linear (2-D) ion trap-Fourier transform ion cyclotron resonance (FTICR) mass spectrometer (ThermoElectron, San Jose, CA) was operated in the data-dependent mode using the TOP10 strategy. For each cycle, one full MS scan acquired on the LTQ FT at high mass resolution was followed by ten MS/MS spectra on the linear ion trap from the ten most abundant ions.

Peptide identification

MS/MS spectra collected from 12 runs were recalibrated and searched using the SEQUEST algorithm (2) against a composite database containing the *Drosophila melanogaster* protein database (R4.3) and its reversed complement based on target and decoy strategy (3) to estimate false positive rate. An initial search were performed by using tryptic specificity; a mass tolerance

of 100 ppm; 2 mis-cleavage sites, a static modification of 71.0371 Da on cysteine; and dynamic modifications of 15.99491 Da (oxidation) on methionine and 8.01420 Da on lysine and 10.00826 Da on arginine residues to account for the presence of both light and heavy peptide. Results were first filtered by in-house software considering the combination of mass tolerance (ppm), peptide length, XCorr and dCn to achieve maximum sensitivity levels while maintaining a <1% false discovery rate.

Peptide and protein quantification

Peptide quantification was performed using an automated method VISTA (4). The quantitative ratios associated with each peptide were accepted if peptides had high VISTA confidence score (≥90). Peptides with the same amino acid sequences were considered unique if they had different charges, different states of methionine oxidation or were from different collections of the 12 fractions. The Grubbs test was used to eliminate outliers in quantification for cases in which multiple peptides were quantified for each protein. Protein ratios were first calculated by averaging the $\log_2$-transformed non-redundant peptide ratios between the heavy isotope labeled (H) and light isotope labeled (L) as $\log_2$ (H/L) and then normalized to the median ratio value to give a population centered on 0 for tabulation. The level of differential protein expression is represented as fold change up- or down-regulated in *miR-8*$^{\Delta/\Delta}$ (H) compared with *w1118* (L) in Supplemental Table S2.

Functional cluster analysis

FlyBase gene identification numbers (FBgn) and UniProt entries of embryonic proteins identified from *in vivo* SILAC were binned according to the H/L protein ratio. Functional cluster analysis was performed on the top 200 differentially expressed proteins, including changes in both up- and down-regulation in the *miR-8*$^{\Delta/\Delta}$ mutant, by web-based tools available on DAVID Bioinformatics Database 6.7 at http://david.abcc.ncifcrf.gov (5, 6). Enrichment of biological processes, which are characterized by gene ontology (GO) terms, protein interactions and more than 40 categories in this database, was calculated against the number of genes that belong to the same category in the entire annotated *Drosophila melanogaster* genome as background. The enrichment score is set as 1.0 at the background level. Terms with a hypergeometric *p* value <0.05 in at least one bin were selected, log transformed, z-transformed, hierarchically clustered to generate heat maps. Resultant clusters of biological processes (minimum = 2 terms/bin) with

the highest median enrichment scores across different bins are shown in the Supplemental Table S1. As a control, 200 randomly generated genes was subjected to the same analysis and we identified no significant enrichment of any functional cluster with a p value < 0.1.

MiRNA target predictions

Prediction of miR-8 targets was performed by TargetScan Fly Release 5.1 (http://www.targetscan.org/fly). The TargetScanS computational algorithm (7) searches for conserved motifs in the 3'UTRs of mRNAs that match the seed region of mature miR-8 (position +2-8:AAUACUG) across 12 sequenced Drosophilid genomes (8, 9). The 3'UTRs of Engrailed (en; CG9015) has one exact match for miR-8 from position +2-8 followed by an A (7-mer 1A).

Figures:

Lu et al., Figure S1

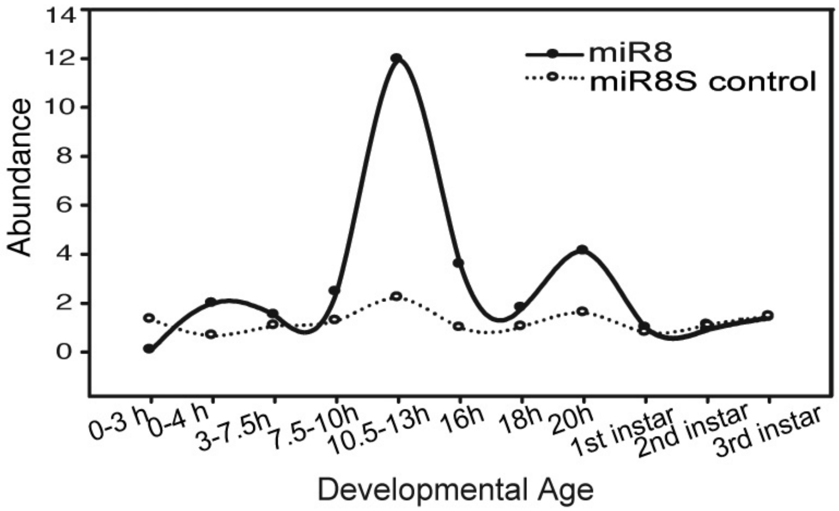

Figure S1. Expression profile of miR-8 during embryonic and larval development analyzed by quantitative RT-PCR. Amplification end products of miR-8 (solid line) and anti-sense miR-8S (dotted line) are plotted along the developmental age of the embryos at the time of collection. Quantification of abundance is performed as described in the Materials and Method section.

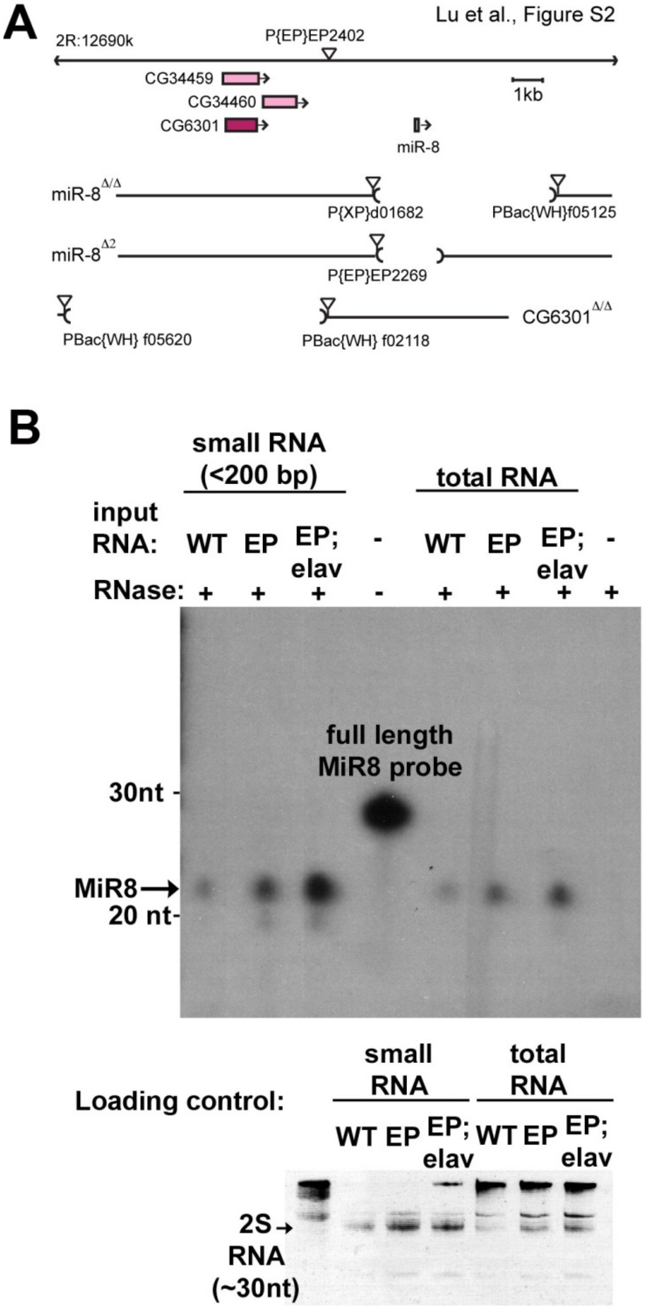

Figure S2. MiR-8 locus and mutants

(A) Schematic representation of different *miR-8* alleles on the right arm of the 2nd chromosome (2R) and the boundaries of their genomic positions as framed by the transposons (inversed open triangles). A number of transposons P{XP}, PBac{WH}, and P{EP} lines each carries a transposable element insertion, in some of which the P- or PBac-element has been modified to carry Gal4 binding sites (UAS), are inserted between CG34460 and miR-8 as annotated in the FlyBase release FB2010_08. Although earlier mRNA/genomic alignment and annotation of CG6301 is replaced by CG34459 and CG34460 in the current release of FlyBase, we use CG6301 here to be consistent with previous miR-8 studies. Scale bar =1 kb. The genomic deletions are verified by genomic PCR and sequencing as described (10) and in Materials and Methods. (B) Detection of mature miR-8 level by RNase protection assay. The EP line P{EP}EP2402 carrying the UAS inserted next to the miR-8 gene was crossed with Elav-Gal4 flies carrying the transcriptional activator Gal4 expressed in post-mitotic neurons to drive the expression miR-8 in *Drosophila* embryos. Thus, misexpression of mature miR-8 can be achieved by driving the P{EP} in this genomic location with a tissue-specific Gal-4 driver.

Lu et al., Figure S3

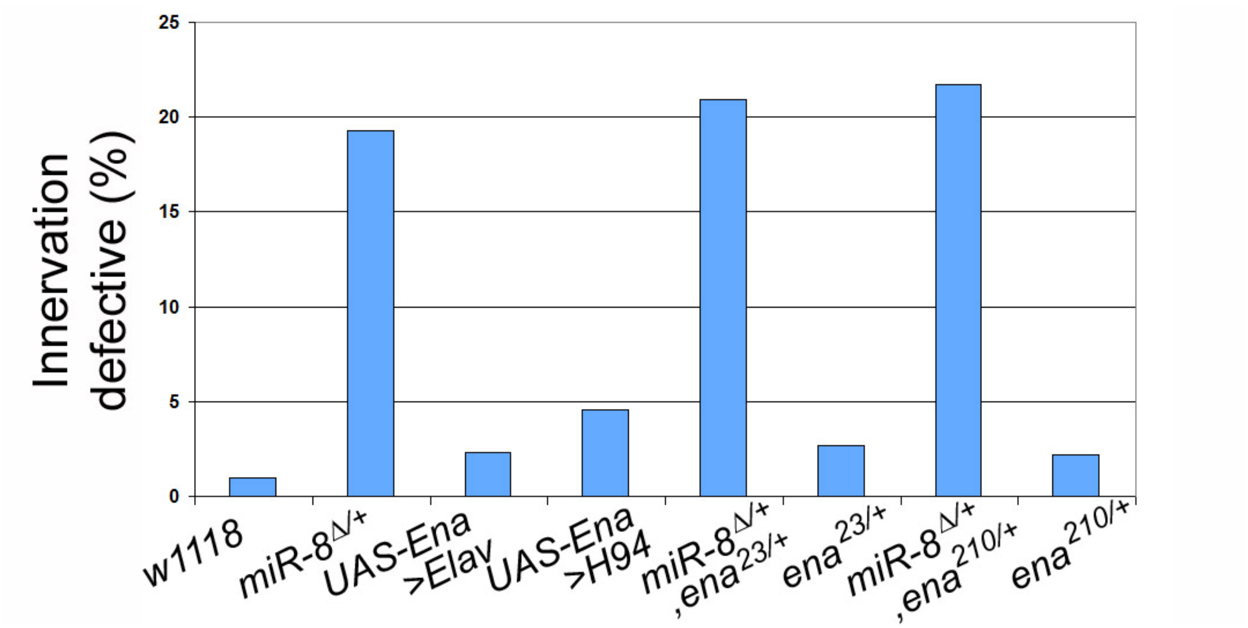

Figure S3. Embryonic miR-8 does not contribute to defective ISNb innervations at muscles m6/m7 cleft via Enabled (Ena)-dependent pathways. The frequency of defective innervation is characterized by the absence of anti-Fas II immunoreactivity or a reduction of more than 2/3 of wild-type length at the m6/m7 cleft by ISNb branch *in situ*. The severity of phenotype is expressed as a percentage of affected hemisegments. Elav-GAL4 drives transgene expression in all post-mitotic neurons and H94-GAL4 targets expression in a subset of embryonic ventral muscles, m4, m6, and m13 (11). In contrast to similar assays performed at 3rd instar larval stage, overexpression of Ena transgene in neither neurons (*UAS-Ena>Elav*, n=195) or target muscles (*UAS-Ena>H94*, n=253) phenocopied the weak m6/m7 innervation in miR-8 heterozygotes (*miR-8*$^{\Delta/+}$). Furthermore, suppression of the physiological function of Ena downstream of miR-8 by disabling EVH1 (*miR-8*$^{\Delta/+}$, *ena*$^{23/+}$, n=181) and truncating EVH2 domain of Ena (*miR-8*$^{\Delta/+}$, *ena*$^{210/+}$, n=97) to prevent binding with its cytoskeletal effector proteins (12) did not rescue the miR-8 phenotype (*miR-8*$^{\Delta/+}$, n=285).

Lu et al., Figure S4

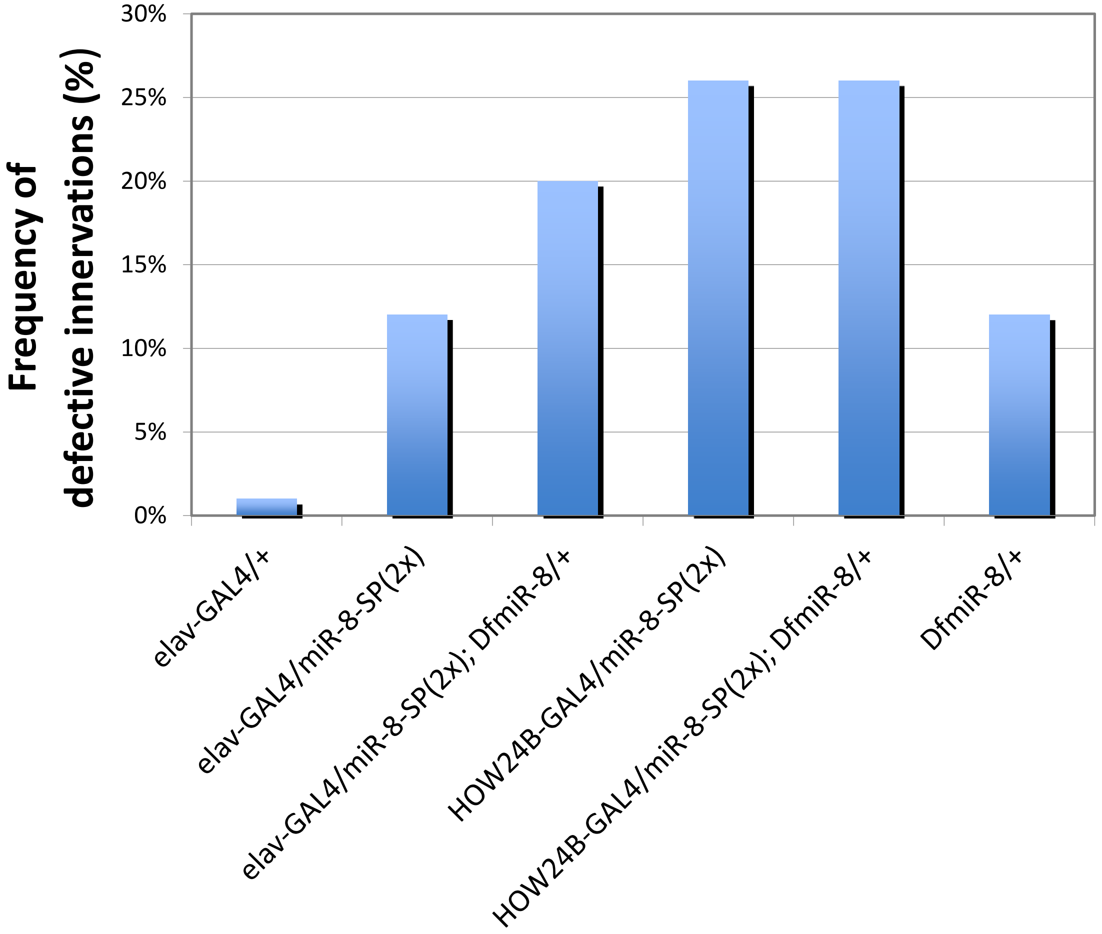

Figure S4. Embryonic miR-8 acts pre- and post-synaptically to promote robust motor axon ISNb innervations at muscles m6/m7 cleft. MiR-8 antagomer UAS-miR-8-SP (13) was driven by Elav-GAL4 to express in all post-mitotic neurons or by HOW24B-GAL4 in all embryonic muscles, and the frequency of defective ISNb innervations was assessed. The severity of phenotype is expressed as a percentage of affected hemisegments with absent anti-Fas II immunostaining in the ISNb axon terminals at muscles m6/m7 cleft. Over expression of miR-8-SP sponge in either neurons (*miR-8-SP(2x)/elav-Gal4*, n=418) or muscles (*miR-8-SP(2x)/HOW24B-Gal4*, n=84) in the *w1118* wild-type background phenocopied *miR-8* null mutant heterozygotes (*DfmiR-8*$^{\Delta/+}$, n=49). Pre-synaptic knock-down of miR-8 in *miR-8* null background moderately augmented the penetrance of innervations defects *(miR-8-SP(2x)/elav-Gal4;DfmiR-8*$^{\Delta/+}$+, n=319) while post-synaptic knock-down of miR-8 did not (*miR-8-SP(2x)/HOW24B-Gal4;DfmiR-8*$^{\Delta/+}$+, n=38).

Tables:

Lu et al., Table S1

Cluster name	Enrichment score	% total candidates	p value
ribosome and translational process	4.76	38.50%	5.80E-05
spectrosome organization	2.67	5.20%	3.20E-04
Rab GTPases	2.27	17.60%	2.40E-05
mitochondrial proton-transporting ATP synthase complex	2.21	26.20%	1.00E-03
heat shock protein	1.82	1.40%	8.40E-03
septate juction	1.65	11.40%	9.30E-04
synapse organization and neuromuscular juction development	1.51	7.60%	3.80E-04
ferric ion transport	1.48	8.60%	7.50E-03
DNA polymerase activity	1.24	7.10%	3.60E-03
proteasome	0.99	5.20%	1.20E-03

Table S1 List of enriched functional clusters dependent on miR-8
Functional clustering of biological processes is ranked by enrichment scores. Only clusters with significant p values ($p < 0.01$) are included in the list. Note that candidates emerged from differential *in vivo* SILAC contain gene ontology (GO) terms that belong to more than one functional cluster and therefore the sum of % total candidates adds up to more than 100%.

Table S2 List of proteins with significant differential expression levels between wild-type and *miR-8* $^{\Delta/\Delta}$ embryos as determined by *in vivo* SILAC.

Protein ID	FlyBase gene ID	# of peptide	Standard deviation	Normalized average log2(H/L)	Fold change in protein expression	Down- or up-regulated in *miR-8 null* embryos
**Mhc-PK**	**FBgn0002741**	17	0.523	-2.78	0.146	down
**His2B:CG17949-PA**	**FBgn0061209**	4	0.1	-2.541	0.172	down
**fit-PA**	**FBgn0038914**	1	0	-2.514	0.175	down
**RpL23-PA**	**FBgn0010078**	2	0.15	-2.281	0.206	down
**CG9894-PB**	**FBgn0031453**	1	0	-2.17	0.222	down
**His4:CG33901-PA**	**FBgn0053901**	3	0.28	-1.928	0.263	down
**CG7768-PA**	**FBgn0036415**	3	0.219	-1.889	0.27	down
**Tm1-PE**	**FBgn0003721**	1	0	-1.778	0.292	down
**RpS12-PA**	**FBgn0014027**	1	0	-1.744	0.298	down
**hoip-PA**	**FBgn0015393**	2	0.429	-1.741	0.299	down
**CG6958-PA**	**FBgn0039004**	1	0	-1.721	0.303	down
**ben-PA**	**FBgn0000173**	1	0	-1.709	0.306	down
**Gs1-PB**	**FBgn0001142**	1	0	-1.628	0.323	down
**RpS14b-PA**	**FBgn0004404**	4	1.89	-1.53	0.346	down
**Fmr1-PE**	**FBgn0028734**	1	0	-1.503	0.353	down
**LanB2-PA**	**FBgn0002528**	1	0	-1.486	0.357	down
**CG11857-PA**	**FBgn0039303**	1	0	-1.483	0.358	down
**RpS15-PB**	**FBgn0034138**	1	0	-1.479	0.359	down
**Hrb87F-PA**	**FBgn0004237**	1	0	-1.461	0.363	down
**CG4225-PA**	**FBgn0038376**	1	0	-1.457	0.364	down
**Pep-PB**	**FBgn0004401**	1	0	-1.444	0.368	down
**RpL29-PB**	**FBgn0016726**	3	0.247	-1.432	0.371	down
**CG6133-PA**	**FBgn0026079**	1	0	-1.425	0.372	down
**Df31-PA**	**FBgn0022893**	5	0.416	-1.377	0.385	down
**Ntf-2-PA**	**FBgn0031145**	1	0	-1.363	0.389	down
**RpS15Aa-PE**	**FBgn0010198**	5	0.201	-1.342	0.394	down
**Dhh1-PA**	**FBgn0011802**	1	0	-1.323	0.4	down
**sle-PA**	**FBgn0037810**	1	0	-1.314	0.402	down
**CG7637-PA**	**FBgn0033548**	1	0	-1.308	0.404	down
**Cpr-PA**	**FBgn0015623**	2	0.815	-1.282	0.411	down
**Vha36-PA**	**FBgn0022097**	1	0	-1.271	0.414	down
**cora-PA**	**FBgn0010434**	1	0	-1.264	0.416	down
**Sod-PA**	**FBgn0003462**	2	0.503	-1.261	0.417	down
**Fas3-PA**	**FBgn0000636**	1	0	-1.253	0.42	down
**Nrg-PB**	**FBgn0002968**	3	0.513	-1.246	0.422	down

Protein ID	FlyBase gene ID	# of peptide	Standard deviation	Normalized average log2(H/L)	Fold change in protein expression	Down- or up-regulated in *miR-8 null* embryos
**Mgstl-PA**	**FBgn0025814**	1	0	-1.243	0.422	down
**beta-Spec-PA**	**FBgn0250788**	10	0.401	-1.238	0.424	down
**l(2)06225-PA**	**FBgn0010612**	1	0	-1.219	0.43	down
**Gnf1-PA**	**FBgn0004913**	1	0	-1.217	0.43	down
**capt-PA**	**FBgn0028388**	2	1.477	-1.216	0.43	down
**CG1910-PA**	**FBgn0022349**	2	0.115	-1.214	0.431	down
**lig-PC**	**FBgn0020279**	2	1.452	-1.178	0.442	down
**hts-PB**	**FBgn0004873**	3	0.47	-1.176	0.442	down
**CG9000-PA**	**FBgn0034176**	1	0	-1.16	0.448	down
**Atpalpha-PA**	**FBgn0002921**	2	0.149	-1.15	0.451	down
**alpha-Spec-PA**	**FBgn0250789**	29	0.645	-1.139	0.454	down
**CG10627-PA**	**FBgn0036298**	1	0	-1.137	0.455	down
**mus209-PB**	**FBgn0005655**	7	0.376	0.874	1.832	up
**Ard1-PC**	**FBgn0036064**	1	0	0.876	1.835	up
**Fer2LCH-PC**	**FBgn0015221**	5	0.194	0.879	1.839	up
**FK506-bp1-PA**	**FBgn0013269**	3	0.387	0.888	1.851	up
**CG31120-PB**	**FBgn0051120**	1	0	0.895	1.86	up
**AnnX-PA**	**FBgn0000084**	1	0	0.9	1.866	up
**CG11526-PB**	**FBgn0035437**	1	0	0.935	1.912	up
**CG17333-PA**	**FBgn0030239**	2	0.295	0.943	1.922	up
**CG10664-PA**	**FBgn0032833**	2	3.273	0.956	1.941	up
**CG9273-PA**	**FBgn0032906**	1	0	0.958	1.942	up
**Hsp27-PA**	**FBgn0001226**	9	0.76	0.966	1.954	up
**cdc2-PA**	**FBgn0004106**	1	0	0.969	1.958	up
**RpS5b-PA**	**FBgn0038277**	1	0	0.973	1.963	up
**Rpn9-PB**	**FBgn0028691**	8	0.472	0.989	1.985	up
**Mapmodulin-PA**	**FBgn0034282**	1	0	1.009	2.013	up
**CG2943-PA**	**FBgn0037530**	6	0.409	1.034	2.048	up
**CG6028-PB**	**FBgn0038924**	1	0	1.094	2.135	up
**coro-PA**	**FBgn0033109**	2	1.035	1.099	2.142	up
**CG6045-PA**	**FBgn0038349**	4	0.37	1.103	2.148	up
**CG3520-PA**	**FBgn0034859**	1	0	1.125	2.182	up
**CG31714-PA**	**FBgn0032180**	1	0	1.143	2.209	up
**Mdh-PA**	**FBgn0029155**	1	0	1.143	2.209	up
**Fdh-PA**	**FBgn0011768**	2	0.095	1.157	2.231	up
**ox-PA**	**FBgn0011227**	1	0	1.161	2.237	up
**Vha100-2-PB**	**FBgn0028670**	1	0	1.168	2.246	up
**CG4278-PA**	**FBgn0014092**	1	0	1.24	2.361	up

Protein ID	FlyBase gene ID	# of peptide	Standard deviation	Normalized average log2(H/L)	Fold change in protein expression	Down- or up-regulated in *miR-8 null* embryos
**CG32210-PA**	**FBgn0052210**	2	0.281	1.316	2.49	up
**CG30359-PA**	**FBgn0050359**	2	1.983	1.331	2.516	up
**piwi-PA**	**FBgn0004872**	1	0	1.39	2.62	up
**p38b-PA**	**FBgn0024846**	1	0	1.392	2.625	up
**regucalcin-PD**	**FBgn0030362**	1	0	1.463	2.756	up
**Cbp80-PA**	**FBgn0022942**	1	0	1.486	2.801	up
**RpS17-PB**	**FBgn0005533**	6	0.342	1.499	2.826	up
**CG2158-PA**	**FBgn0033264**	1	0	1.506	2.841	up
**RpL36-PA**	**FBgn0002579**	3	0.427	1.526	2.881	up
**CG9643-PA**	**FBgn0031485**	1	0	1.608	3.048	up
**l(1)G0156-PA**	**FBgn0027291**	1	0	1.715	3.282	up
**Tsf1-PA**	**FBgn0022355**	7	0.319	1.914	3.77	up

Table S2 List of proteins with differential expression levels in wild-type and *miR-8* $^{\Delta/\Delta}$ embryos determined by *in vivo* SILAC (continued from previous pages)

References:

1. Hartenstein V, editor. Atlas of *Drosophila* Development. Cold Spring Harbor: Cold Spring Harbor Laboratory Press; 1993.
2. Eng JK, McCormack AL, Yates III JR. An approach to correlate tandem mass spectral data of peptides with amino acid sequences in a protein database. Journal of the American Society for Mass Spectrometry. 1994;5(11):976-89.
3. Elias JE, Gygi SP. Target-decoy search strategy for increased confidence in large-scale protein identifications by mass spectrometry. Nature Methods. 2007;4:207-14.
4. Bakalarski CE, Elias JE, Villén J, Haas W, Gerber SA, Everley PA, et al. The impact of peptide abundance and dynamic range on stable-isotope-based quantitative proteomic analyses. J Proteome Res. 2008;7(11):4756-65.
5. Dennis G, Sherman B, Hosack D, Yang J, Gao W, Lane HC, et al. DAVID: Database for Annotation, Visualization, and Integrated Discovery. Genome Biology. 2003 2003;4(5):R60.
6. Huang DW, Sherman BT, Lempicki RA. Systematic and integrative analysis of large gene lists using DAVID bioinformatics resources. Nat Protocols. 2008;4(1):44-57.
7. Lewis BP, Burge CB, Bartel DP. Conserved seed pairing, often flanked by adenosines, indicates that thousands of human genes are microRNA targets. Cell. 2005;120(1):15-20.
8. Ruby JG, Stark A, Johnston WK, Kellis M, Bartel DP, Lai EC. Evolution, biogenesis, expression, and target predictions of a substantially expanded set of Drosophila microRNAs. Genome Biol. 2007 December 1, 2007;17(12):1850-64.

9. Kheradpour P, Stark A, Roy S, Kellis M. Reliable prediction of regulator targets using 12 Drosophila genomes. Genome Biol. 2007 December 1, 2007;17(12):1919-31.
10. Karres JS, Hilgers V, Carrera I, Treisman J, Cohen SM. The Conserved microRNA MiR-8 Tunes Atrophin Levels to Prevent Neurodegeneration in Drosophila. Cell. 2007;131(1):136-45.
11. Inaki M, Shinza-Kameda M, Ismat A, Frasch M, Nose A. Drosophila Tey represses transcription of the repulsive cue Toll and generates neuromuscular target specificity. Development. 2010;137(13):2139-46.
12. Ahern-Djamali SM, Comer AR, Bachmann C, Kastenmeier AS, Reddy SK, Beckerle MC, et al. Mutations in Drosophila enabled and rescue by human vasodilator-stimulated phosphoprotein (VASP) indicate important functional roles for Ena/VASP homology domain 1 (EVH1) and EVH2 domains. Molecular Biology of the Cell. 1998;9(8):2157-71.
13. Bejarano F, Bortolamiol-Becet D, Dai Q, Sun K, Saj A, Chou Y-T, et al. A genome-wide transgenic resource for conditional expression of Drosophila microRNAs. Development. 2012 August 1, 2012;139(15):2821-31.
